# Increased risk of hypocalcemia with decreased kidney function in patients prescribed bisphosphonates based on real-world data from the MID-NET^®^ in Japan: a new-user cohort study

**DOI:** 10.1186/s12882-024-03553-7

**Published:** 2024-04-15

**Authors:** Tomoaki Hasegawa, Maki Komamine, Chieko Ishiguro, Haruka Motomura, Kazuhiro Kajiyama, Takahiro Nonaka, Yuki Nakazato, Ryota Kimura, Harumi Maniwa, Toyotaka Iguchi, Naoya Horiuchi, Yoshiaki Uyama

**Affiliations:** 1 Office of Medical Informatics and Epidemiology, Pharmaceuticals and Medical Devices Agency, Kasumigaseki 3-3-2, Chiyoda-Ku, Tokyo, 100-0013 Japan; 2 Office of Pharmacovigilance I, Pharmaceuticals and Medical Devices Agency, Tokyo, Japan; 3 Office of Pharmacovigilance II, Pharmaceuticals and Medical Devices Agency, Tokyo, Japan; 4 Office of Regulatory Science Research, Pharmaceuticals and Medical Devices Agency, Tokyo, Japan; 5 Present address: Section of Clinical Epidemiology, Department of Data Science, Center for Clinical Sciences, National Center for Global Health and Medicine, Tokyo, Japan; 6 Present address: National Institutes of Biomedical Innovation, Health and Nutrition, Osaka, Japan; 7 Present address: Department of Health and Medical Innovation, Graduate School of Medicine, Osaka Metropolitan University, Osaka, Japan

**Keywords:** Bisphosphonates, Hypocalcemia, Renal impairment, Pharmacoepidemiology, Real-world evidence

## Abstract

**Background:**

In the post-marketing stage, cases of hypocalcemia associated with bisphosphonate preparations (BPs) have been reported in patients with decreased kidney function, despite warning against use of BPs in such patients in the package insert (PI) of Japan. The purpose of this study was to investigate the safety of BPs in patients with decreased kidney function.

**Methods:**

The cohort study was conducted in patients with osteoporosis and newly prescribed bisphosphonate utilizing real-world data from MID-NET^®^ in Japan. The adjusted hazard ratios (aHRs) for hypocalcemia (a corrected serum Ca level < 8.00 mg/dL) relative to the normal group were calculated in each decreased kidney function group (mild, moderate or severe group).

**Results:**

A total of 14,551 patients were included in the analysis, comprising 2,601 (17.88%) with normal (eGFR ≥ 90 mL/min/1.73m^2^), 7,613 (52.32%) with mild (60 ≤ eGFR < 90 mL/min/1.73m^2^), 3,919 (26.93%) with moderate (30 ≤ eGFR < 60 mL/min/1.73m^2^), and 418 (2.87%) with severe kidney function (eGFR < 30 mL/min/1.73m^2^). The aHRs (95% confidence interval) for hypocalcemia were 1.85 (0.75–4.57), 2.30 (0.86–6.21), and 22.74 (8.37–61.78) in the mild, moderate, and severe groups, respectively. The increased risk of hypocalcemia depending on kidney function was also observed even when calculating the aHR for each specific BP such as alendronate sodium hydrate, minodronic acid hydrate, and sodium risedronate hydrate. Furthermore, similar results were obtained in the sensitivity analysis by altering the outcome definition to a 20% or more reduction in corrected serum Ca level from the baseline, as well as when focusing on patients with more than one laboratory test result per 30 days during the follow-up period.

**Conclusions:**

These findings suggest that the risk of hypocalcemia during BP prescription is higher in patients with decreased kidney function, particularly those with severely decreased kidney function. The quantitative real-world evidence on the safety risk of BPs obtained in this study has led to the PI revision describing a relationship between hypocalcemia risk and decreased kidney function as a regulatory action in Japan and will contribute to promoting the proper use of BPs with appropriate risk management in clinical practice.

**Supplementary Information:**

The online version contains supplementary material available at 10.1186/s12882-024-03553-7.

## Background

Bisphosphonate preparations (BPs) are widely used as the first-line drug for osteoporosis other than early postmenopausal osteoporosis [1–3]. Actually, the use of BPs in patients with severely decreased kidney function has been alerted in the package insert (PI) on the "Contraindications" section of etidronate disodium [4], sodium risedronate hydrate [5, 6], and zoledronic acid hydrate [7], and on the "Precautions Concerning Patients with Specific Backgrounds" section of many BPs [8]. This precautionary stance arises from the absence of clinical BP use in such patients and potentially increased risk inferred from the renal excretion pathway of BPs [9]. Additionally, hypocalcemia is known as an adverse reaction of BPs [9–12] and precaution for hypocalcemia was commonly included in the PI for BPs except etidronate disodium [4–8], although no information about a relationship between hypocalcemia risk and decreased kidney function was included.

Regarding the situation at post-marketing in Japan, results in drug use surveys and spontaneous adverse reaction reports indicate instances of hypocalcemia during BPs administration in patients with decreased kidney function [13]. It suggests the prescription of BPs to patients with decreased kidney function even in the absence of quantitative safety information regarding BPs in clinical practice in Japan.

Consequently, the Pharmaceuticals and Medical Devices Agency (PMDA) has decided to undertake a pharmacoepidemiological study to comprehend the safety characteristics of BPs in patients with decreased kidney function based on the risk of hypocalcemia as an indicator within a real-world setting.

## Methods

### Database

Real-world data from MID-NET^®^, a reliable and valuable database in Japan [14, 15], were used for analysis in this study because MID-NET^®^ stores electronic medical records, administrative claim data, and diagnosis procedure combination (DPC) data for over 6.05 million patients (as of December 2022) in cooperation with 10 healthcare organizations, including 23 university hospitals and regional core hospitals. The study period spanned from January 1, 2009, to March 31, 2019.

Utilizing MID-NET^®^ for this study was approved on February 19, 2020, through a discussion by the expert committee of MID-NET^®^ [16] and the actual data extraction from MID-NET^®^ for analysis was carried out in the week of May 22, 2020.

### Cohort and study design

**Fig. 1:**
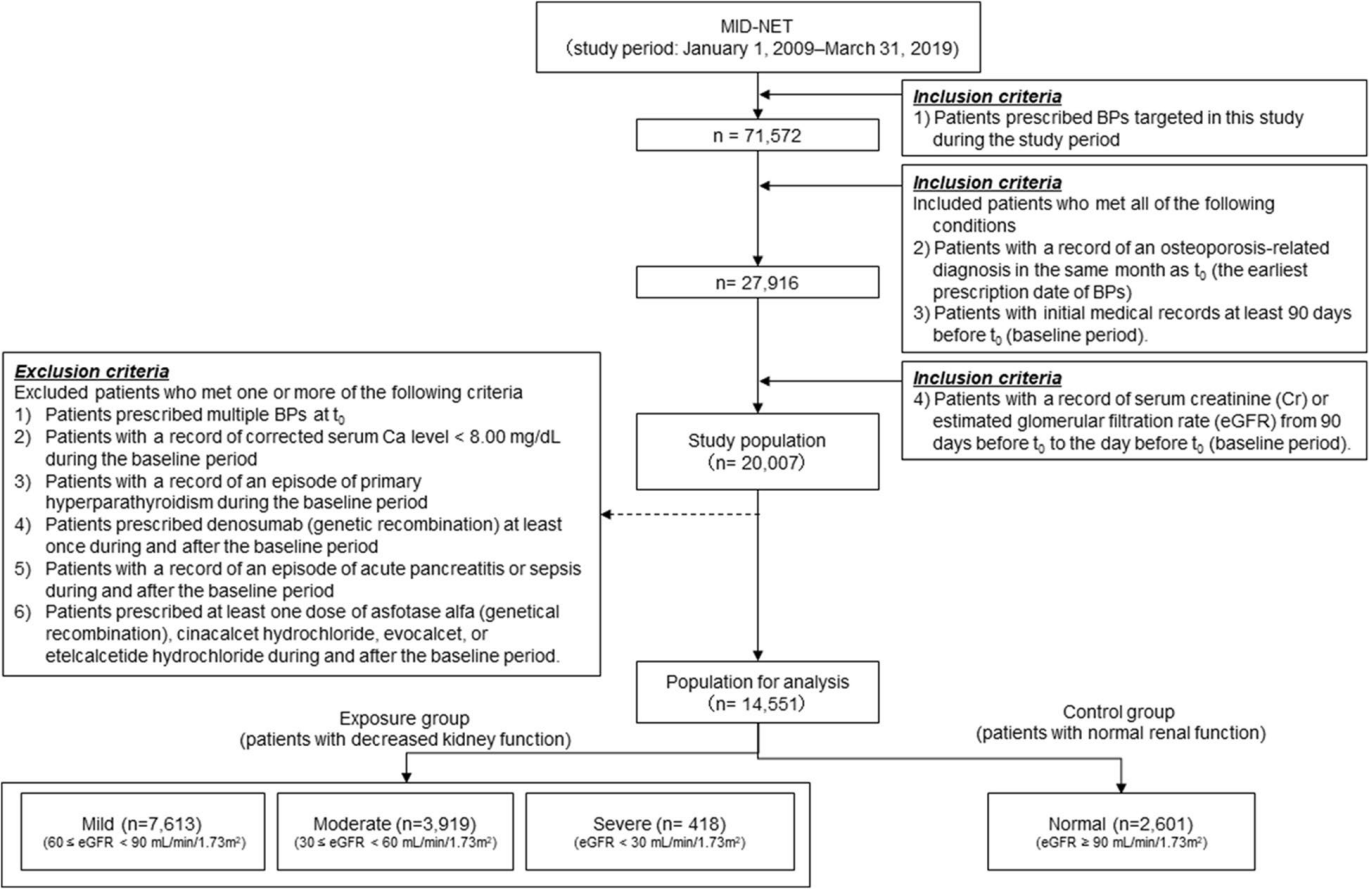
Flow chart for patient selection

In this study, a new-user cohort design was selected for considering the degree of renal dysfunction and abnormal serum calcium (Ca) levels after BP administration. Specifically, as shown in Fig. 1, patients who met all of the following conditions were included in this study; 1) patients prescribed BPs targeted in this study during the study period, 2) patients with a record of an osteoporosis-related diagnosis in the same month as t_0_ (the earliest prescription date of BPs), 3) patients with initial medical records at least 90 days before t_0_, and 4) patients with a record of serum creatinine (Cr) or estimated glomerular filtration rate (eGFR) from 90 days before t_0_ to the day before t_0_ (baseline period). For the analysis, patients who met one or more of the following criteria were excluded to select an appropriate population without a higher risk of hypocalcemia; 1) patients prescribed multiple BPs at t_0_, 2) patients with a record of corrected serum Ca level < 8.00 mg/dL during the baseline period, 3) patients with a record of an episode of primary hyperparathyroidism during the baseline period, 4) patients prescribed denosumab (genetical recombination) at least once during and after the baseline period, 5) patients with a record of an episode of acute pancreatitis or sepsis during and after the baseline period, and 6) patients prescribed at least one dose of asfotase alfa (genetical recombination), cinacalcet hydrochloride, evocalcet, or etelcalcetide hydrochloride during and after the baseline period (see Additional file 1 for more details of this study design).

The target BPs in this study included all BPs (not only oral preparations but also intravenous preparations) with an indication for osteoporosis marketed in Japan during the study period; i.e., alendronate sodium hydrate, etidronate disodium, ibandronate sodium hydrate, minodronic acid hydrate, sodium risedronate hydrate, and zoledronic acid hydrate.

### Definition of exposure

In order to evaluate the relationship between the incidence of hypocalcemia and decreased renal function, renal function was stratified into four categories based on eGFR value: normal (eGFR ≥ 90 mL/min/1.73m^2^), mild (60 mL/min/1.73m^2^ ≤ eGFR < 90 mL/min/1.73m^2^), moderate (30 mL/min/1.73m^2^ ≤ eGFR < 60 mL/min/1.73m^2^), severe (eGFR < 30 mL/min/1.73m^2^). In cases where eGFR values were not recorded, the values were calculated from serum Cr values using the following formula widely used in Japan [17, 18]: 194 × serum creatinine^−1.094^ × age^−0.287^ (× 0.739 for women) (mL/min/1.73m^2^). In instances where multiple eGFR values were available on the same day, the mean eGFR value was used. The baseline eGFR value was determined as the mean of the two eGFR values closest to t_0_ during the baseline period. If only one examination was performed during the baseline period, that single value was used as the baseline eGFR. No data imputations for missing values were made in calculating eGFR.

### Definition of prescription and follow-up periods

The prescription period encompassed the start date (t_0_) and duration of the prescription with a gap and a grace period, in consideration of prescription interval for each BP and a deviation from the scheduled visit time, etc. The gap and the grace periods were equally set based on the information in the PI; i.e., 14 days for BPs prescribed either once a week or once a day, 56 days for BPs prescribed once every 4 weeks, 60 days for BPs prescribed once a month, and 90 days for BPs prescribed once a year. Consequently, two prescriptions for the same drug were considered continuous if the latter prescription date fell within the gap period of the former prescription date.

The follow-up period commenced at t_0_ and concluded at the earliest date of the following; 1) the end date of the prescription period, 2) the day before the start date of another different BP prescription from t_0_, 3) the date of changing renal function category defined as the second date of two consecutive changes of a different category from the baseline, or 4) the end date of the study period (March 31, 2019).

### Definition of outcome

"Hypocalcemia" was defined as a corrected serum Ca level < 8.00 mg/dL, as per Payne's equation, a widely employed method in Japan [19, 20]. If multiple values were recorded on the same day, the minimum serum Ca value and the maximum albumin (Alb) value were used. To determine the corrected serum calcium level, the serum Alb value measured within 14 days of the serum Ca measurement was selected, with preference given to the value closest to the date of serum Ca measurement. No data imputations for missing values were made in calculating serum Ca value.

### Definition of covariates

The covariates used in this study included gender (male/female), age (age < 65 years, 65 years to < 75 years, 75 years and older), and complications (hypoparathyroidism, vitamin D deficiency, magnesium disorders) as well as concomitant drugs (elcatonin, steroids excluding topical preparations, calcium preparations excluding topical preparations, vitamin D preparations excluding topical preparations, sorafenib tosilate, lenvatinib mesilate, vandetanib, enviomycin sulfate, and monobasic sodium phosphate monohydrate/dibasic sodium phosphate anhydrous) among diseases and drugs known to be associated with the risk of hypocalcemia [8, 21, 22]. Data at t_0_ for sex and age, and at the baseline period for complications and concomitant drugs were used for analysis.

### Statistical analysis

Patient background data, including covariates, each active ingredient of BPs at t_0_, concomitant drugs known to cause osteoporosis during the follow-up period, and the calendar year of t_0_ were tabulated.

The incidence rate of hypocalcemia (/patient-year) in each group, the crude hazard ratio (cHR) and adjusted hazard ratio (aHR; with adjustment for the covariates described above) of each group to the normal group (Cox proportional hazards model) were calculated. These analyses were also performed for each active ingredient of BPs at t_0_.

In addition, sensitivity analysis was conducted by changing the outcome definition from “corrected serum Ca level < 8.00 mg/dL” in the primary analysis to “20% or more reduction of corrected serum Ca level from the baseline.” The baseline serum Ca level was defined as the value closest to t_0_ among the corrected serum Ca values in the baseline period. Furthermore, to check the impacts of the lack of laboratory tests during the BP prescription period, the same analysis as the primary was conducted only in patients with more than one laboratory test result per 30 days during the follow-up period. In this population, the median (first quartile, third quartile) frequency for laboratory tests was 1.7 (1.4, 3.2) for the normal, 1.5 (1.4, 3.2) for the mild, 1.8 (1.4, 3.8) for the moderate, and 2.2 (1.4, 5.6) for the severe groups. The aHRs were also calculated when dialysis patients who had a record of dialysis before t_0_ were excluded from the cohort of the primary analysis.

SAS version 9.4 (SAS Institute, Cary, NC, USA) was used for all analysis.

## Results

### Cohort

During the study period, 71,572 patients were prescribed BPs targeted in this study. Of those, 14,551 patients were included for analysis after applying all inclusion and exclusion criteria (Fig. 1). The number of patients in each group was 2,601 (17.88%) for the normal, 7,613 (52.32%) for the mild, 3,919 (26.93%) for the moderate, and 418 (2.87%) for the severe groups.

The patient backgrounds are summarized in Table 1. No major differences among the groups were observed, except for a higher proportion of elderly patients in the moderate and severe groups, and greater number of steroid prescription in the normal group. The most frequently prescribed BP in this study was alendronate sodium hydrate (53.44–64.59%), followed by sodium risedronate hydrate (19.62–30.03%) and minodronic acid hydrate (14.35–16.77%).

**Table 1:**
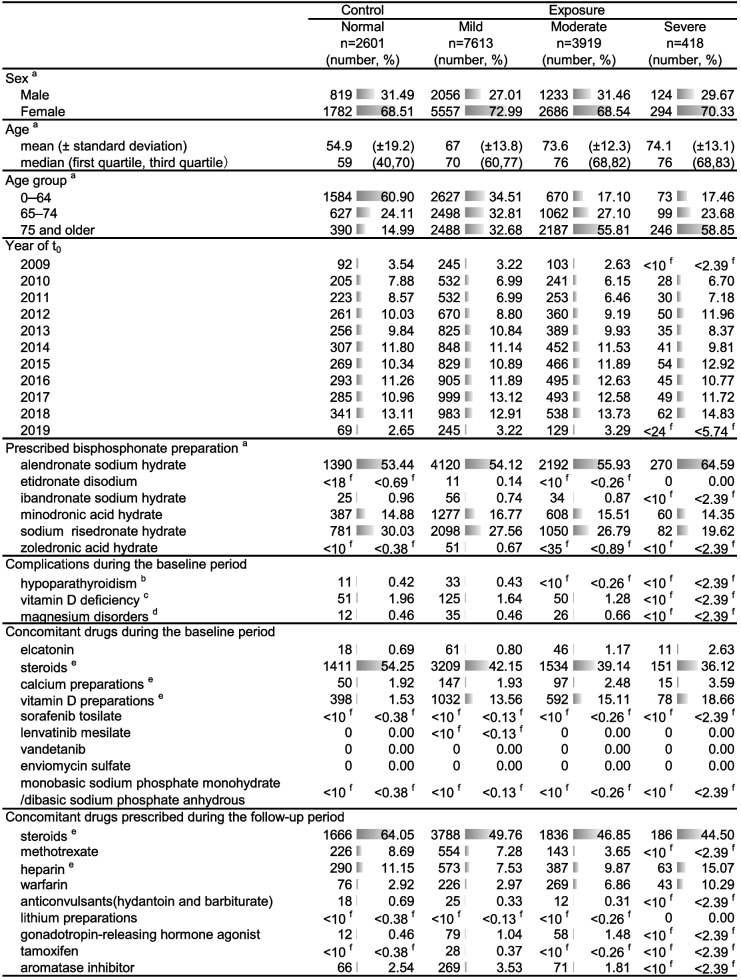
Patients backgrounds

^a^at t_0_

^b^defined based on ICD-10 codes of E200, E201, E208, E209 and E892

^c^defined based on ICD-10 codes of E550, E559 and M8339

^d^defined based on ICD-10 codes of E612, E834, R790 and T568

^e^Excluding topical preparations

^f^When the number of patients was < 10, an aggregated value was presented based on the MID-NET^®^ publication rule, so that the specific number could not be identified

### Risk comparison of hypocalcemia among patients with different categories of decreased kidney functions

**Fig. 2:**
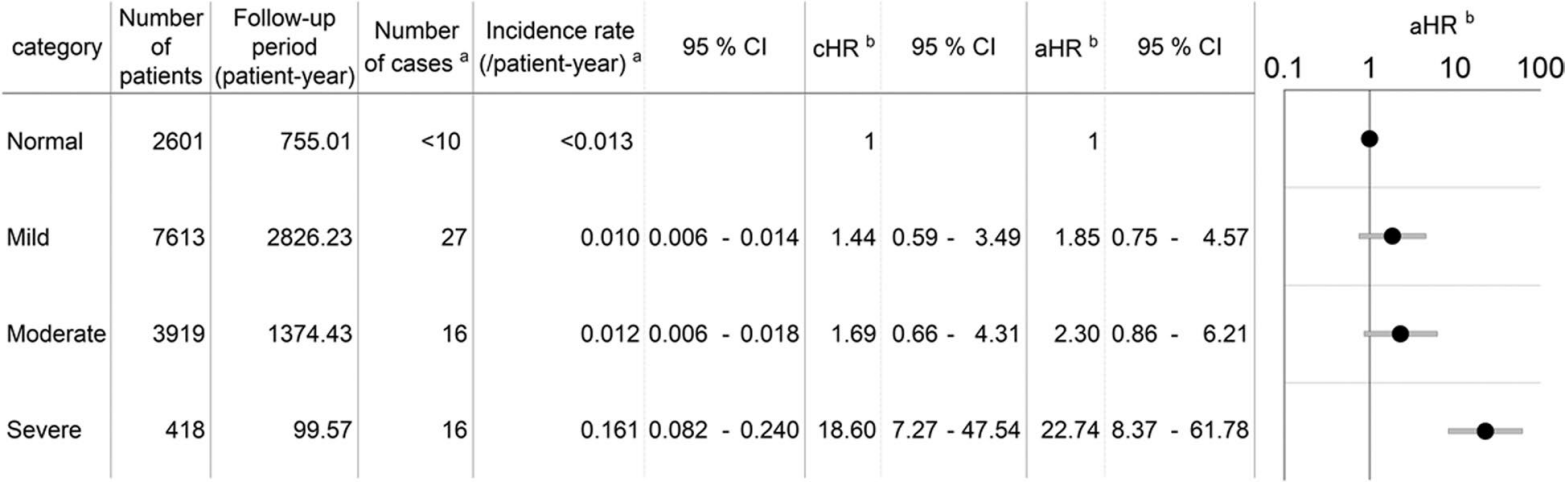
Hazard ratios for hypocalcemia in each group of decreased kidney function (vs normal group). aHR: adjusted hazard ratio, cHR: crude hazard ratio, CI: confidence interval. ^a^When the number of patients was < 10, an aggregated value was presented based on the MID-NET^®^ publication rule, so that a specific number could not be identified. ^b^aHR and cHR were calculated based on the Cox proportional hazards model and aHR was adjusted with the covariates (see “Methods”)

As shown in Fig. 2, the incidence rates of hypocalcemia (corrected serum Ca level < 8.00 mg/dL) were approximately 0.01 for the mild and moderate groups and 0.161 for the severe group. The aHRs (95% confidence interval (CI)) for hypocalcemia relative to the normal group were 1.85 (0.75–4.57), 2.30 (0.86–6.21), and 22.74 (8.37–61.78) in the mild, moderate, and severe groups, respectively. Importantly, the aHR demonstrated a consistent increase in declining kidney function, and the increased risk observed in the severe group was statistically significant. The median time from t_0_ to cause hypocalcemia was 15 days with the first-third quartiles of 6–43 days with similar distribution in all groups.

The increased risk of hypocalcemia (corrected serum Ca level < 8.00 mg/dL) depending on kidney function was also observed even when calculating aHR for each BP. For example, aHRs (95% CI) in the severe group were 16.03 (4.68–54.96) for alendronate sodium hydrate, 40.21 (3.75–430.64) for minodronic acid hydrate, and 23.03 (1.89–280.35) for sodium risedronate hydrate, although no cases of hypocalcemia in the severe group were observed for other BPs due to the limited sample size (see Additional file 2 for other aHRs of each BP).

In the sensitivity analysis by changing the outcome definition to 20% or more reduction in corrected serum Ca level from the baseline, the aHRs (95% CI) (vs normal group, *n* = 1,848) were 0.67 (0.16–2.86) for the mild (*n* = 5,225), 2.25 (0.57–8.94) for the moderate (*n* = 2,700), and 22.89 (5.87–89.21) for the severe (*n* = 338) groups. It should be noted that 4,440 patients in the cohort were excluded for this analysis due to unavailability of serum Ca level during the baseline period.

Additionally, on the analysis targeted only for patients with more than one laboratory test result per 30 days during the follow-up period, the aHRs (95% CI) (vs normal group, *n* = 818) were 2.20 (0.83–5.85) for the mild (*n* = 1,982), 1.95 (0.68–5.61) for the moderate (*n* = 1,224), and 11.34 (3.97–32.36) for the severe (*n* = 217) groups. Furthermore, when dialysis patients were excluded, the aHRs (95% CI) (vs normal group, *n* < 2,601) were 1.81 (0.73–4.49) for the mild (*n* = 7,599), 2.10 (0.77–5.72) for the moderate (*n* = 3,899), and 21.36 (7.75–58.85) for the severe (*n* = 397) groups.

## Discussion

This study examined the characteristics of hypocalcemia during BP prescription. The incidence of hypocalcemia observed in this study was generally low and comparable to the percentage (less than a few percentage; ~ 5%) in the PI of BPs in Japan. BP prescription to patients with severely decreased kidney function have not been generally recommended because of insufficient safety data and the potentially increased risk of adverse events, such as hypocalcemia in these patients [2, 23, 24]. This study provides quantitative data regarding the increased risk of hypocalcemia in patients with decreased kidney function who were prescribed BPs. The magnitude of the increased risk during BP prescription was correlated with the degree of decreased kidney function, with a notably higher risk observed in patients with severely decreased kidney function. The increased risk in the severe group was substantiated by the results of sensitivity analysis where the outcome definition was changed to “20% or more reduction of corrected serum Ca level from the baseline.”

In addition, the similar increased risks of hypocalcemia depending on kidney function were also observed when analyzing only patients with more than one laboratory test result per 30 days during the follow-up period. This result suggests that the increased risk was associated with decreased kidney function rather than variations in the frequency of laboratory tests for serum Ca levels among the groups, such as more frequent in the severe group and less frequent in the normal, the mild or moderate groups. The presence of dialysis patients did not also significantly affect the study results, because the similar increased risks to the primary analysis were observed even when dialysis patients were excluded from the analysis. Differences in patient backgrounds among the groups were also unlikely to affect the results, because at least the backgrounds between the moderate and severe groups were similar including age distribution and the percentage of steroid prescription.

Furthermore, similar increased risk of hypocalcemia was identified across different BPs, such as alendronate sodium hydrate, minodronic acid hydrate, and sodium risedronate hydrate. These results suggest that the increased risk of hypocalcemia may be a common characteristic of BPs, supported by their shared pharmacological action of inhibiting bone resorption through osteoclast apoptosis [9]. Recent report also highlight a higher risk of hypocalcemia in patients with decreased kidney function prescribed denosumab or bisphosphonate [25]. Our finding supports those results through pharmacoepidemiological methods analyzing real-world data. The reason for different incidence of hypocalcemia between the studies could be due to the study condition and different data source including different definition of hypocalcemia and different target of BPs (all preparations in our study vs. only oral preparation in the other study, etc.).

As described above, this study delivered real-world evidence regarding the increased risk of hypocalcemia during BP prescription that is useful in clinical practice for promoting proper use of BPs with appropriate risk management.

The strength of this study was the utilization of longitudinal laboratory test results of serum Ca as an outcome of hypocalcemia from MID-NET^®^, a reliable database [14, 15]. However, as a limitation, results may be affected by severity of decreased kidney function itself [26] as well as other potential confounders such as changes of parathyroid function [21, 22] during the follow-up period, a severity of complications other than kidney function and other concomitant drugs not adjusted in this study.

Building on the findings of this study and other relevant information, including case reports and related literature, the safety assessment by the PMDA prompted the Ministry of Health, Labour and Welfare to revise the PI of BPs in January 2023. In this revision more information about the relationship between hypocalcemia risk and decreased kidney function, especially the higher risk of hypocalcemia in patients with severely decreased kidney function, was newly added to inform health care professionals of this risk [27–32].

## Conclusion

The risk of hypocalcemia during BP prescription was found to be higher in patients with decreased kidney function, particularly those with severely decreased kidney function. The quantitative real-world evidence regarding the safety risk of BPs obtained in this study has led to the revision of the PI as a regulatory action in Japan and will contribute to promoting the proper use of BPs with appropriate risk management in clinical practice.

### Supplementary Information

**Supplementary Material 1.**

**Supplementary Material 2.**

## Data Availability

The dataset generated in the study is not publicly available due to the terms of use for MID-NET^®^ to which we adhered when conducting this study; the accessibility of the dataset used for this analysis is restricted to specific authors including the corresponding author in a predetermined secure environment. No outside researchers are allowed to access the dataset.

## Abbreviations

aHR: Adjusted hazard ratio
Alb: Albumin
BP: Bisphosphonate preparation
Ca: Calcium
cHR: Crude hazard ratio
CI: Confidence interval
Cr: Creatinine
DPC: Diagnosis procedure combination
eGFR: Estimated glomerular filtration rate
HR: Hazard ratio
PI: Package insert
PMDA: Pharmaceuticals and Medical Devices Agency

## References

[1] Reid IR (2015). Short-term and long-term effects of osteoporosis therapies. Nat Rev Endocrinol.

[2] Fukagawa M, Yokoyama K, Koiwa F, Taniguchi M, Shoji T, Kazama JJ, Komaba H, Ando R, Kakuta T, Fujii H (2013). Clinical practice guideline for the management of chronic kidney disease-mineral and bone disorder. Ther Apher Dial.

[3] Suzuki Y, Nawata H, Soen S, Fujiwara S, Nakayama H, Tanaka I, Ozono K, Sagawa A, Takayanagi R, Tanaka H (2014). Guidelines on the management and treatment of glucocorticoid-induced osteoporosis of the Japanese Society for Bone and Mineral Research: 2014 update. J Bone Miner Metab.

[4] The package insert of Didronel^®^ Tablets [in Japanese]. [https://www.pmda.go.jp/PmdaSearch/iyakuDetail/ResultDataSetPDF/400093_3999010F1025_2_22]. Accessed 2 Apr 2024.

[5] The package insert of Actonel^®^ Tablets 2.5mg [in Japanese]. [https://www.pmda.go.jp/PmdaSearch/iyakuDetail/ResultDataSetPDF/111890_3999019F1026_2_20]. Accessed 2 Apr 2024.

[6] The package insert of BENET^®^ Tablets 2.5mg [in Japanese]. [https://www.pmda.go.jp/PmdaSearch/iyakuDetail/ResultDataSetPDF/400256_3999019F1034_1_29]. Accessed 2 Apr 2024.

[7] The package insert of Reclast^®^ for i.v. infusion 5mg [in Japanese]. [https://www.pmda.go.jp/PmdaSearch/iyakuDetail/ResultDataSetPDF/100898_3999423A4027_1_09]. Accessed 2 Apr 2024.

[8] Pharmaceuticals and Medical Devices Agency, Information search for new drugs, such as review reports, package inserts, and other related infromation. [in Japanese]. [https://www.pmda.go.jp/PmdaSearch/iyakuSearch/]. Accessed 2 Apr 2024.

[9] Nolin T, Friedman P. Chapter 52 Agents affecting mineral ion homeostasis and bone turnover. In: Goodman & Gilman's, The pharmacological basis of therapeutics, 14th ed. New York, USA: Mc Graw Hill; 2023.

[10] Papapetrou PD (2009). Bisphosphonate-associated adverse events. Hormones (Athens).

[11] Zuradelli M, Masci G, Biancofiore G, Gullo G, Scorsetti M, Navarria P, Tancioni F, Berlusconi M, Giordano L, Santoro A (2009). High incidence of hypocalcemia and serum creatinine increase in patients with bone metastases treated with zoledronic acid. Oncologist.

[12] Reid IR (2015). Efficacy, effectiveness and side effects of medications used to prevent fractures. J InternMed.

[13] Pharmaceuticals and Medical Devices Agency. Japanese Adverse Drug Event Report database [in Japanese]. [https://www.pmda.go.jp/safety/info-services/drugs/adr-info/suspected-adr/0005.html]. Accessed 2 Apr 2024.

[14] Yamaguchi M, Inomata S, Harada S, Matsuzaki Y, Kawaguchi M, Ujibe M, et al. Establishment of the MID-NET^®^ medical information database network as a reliable and valuable database for drug safety assessments in Japan. Pharmacoepidemiol Drug Saf. 2019;28:1395–404. 10.1002/pds.4879 PMC6851601 31464008

[15] Yamada K, Itoh M, Fujimura Y, Kimura M, Murata K, Nakashima N, et al. The utilization and challenges of Japan’s MID-NET^®^ medical information database network in post-marketing drug safety assessments: a summary of pilot pharmacoepidemiological studies. Pharmacoepidemiol Drug Saf. 2019;28:601–8. 10.1002/pds.4777 30945387

[16] Pharmaceuticals and Medical Devices Agency. Information for studies approved through a discussion by the expert committee of MID-NET^®^ [in Japanese]. [https://www.pmda.go.jp/safety/mid-net/0010.html]. Accessed 2 Apr 2024.

[17] Matsuo S, Imai E, Horio M, Yasuda Y, Tomita K, Nitta K, Yamagata K, Tomino Y, Yokoyama H, Hishida A (2009). Revised equations for estimated GFR from serum creatinine in Japan. Am J Kidney Dis.

[18] Japanese Society of Nephrology (2018). Evidence-based clinical practice guideline for CKD 2018. Jpn J Nephrol.

[19] Payne RB, Carver ME, Morgan DB (1979). Interpretation of serum total calcium: effects of adjustment for albumin concentration on frequency of abnormal values and on detection of change in the individual. J Clin Pathol.

[20] Payne RB, Little AJ, Williams RB, Milner JR (1973). Interpretation of serum calcium in patients with abnormal serum proteins. Br Med J.

[21] Fong J, Khan A (2012). Hypocalcemia: updates in diagnosis and management for primary care. Can Fam Physician.

[22] Fukumoto S, Namba N, Ozono K, Yamauchi M, Sugimoto T, Michigami T, Tanaka H, Inoue D, Minagawa M, Endo I (2008). Causes and differential diagnosis of hypocalcemia–recommendation proposed by expert panel supported by ministry of health, labour and welfare. Japan Endocr J.

[23] West SL, Patel P, Jamal SA (2015). How to predict and treat increased fracture risk in chronic kidney disease. J Intern Med.

[24] Nitta K, Yajima A, Tsuchiya K (2017). Management of osteoporosis in chronic kidney disease. Intern Med.

[25] Cowan A, Jeyakumar N, McArthur E, Fleet JL, Kanagalingam T, Karp I, Khan T, Muanda FT, Nash DM, Silver SA (2023). Hypocalcemia risk of denosumab across the spectrum of kidney disease: a population-based cohort study. J Bone Miner Res.

[26] KDIGO 2017 Clinical Practice Guideline Update for the Diagnosis, Evaluation, Prevention, and Treatment of Chronic Kidney Disease–Mineral and Bone Disorder (CKD-MBD). Kidney Int Suppl. 2017;7:1–59. https://kdigo.org/wp-content/uploads/2017/02/2017-KDIGO-CKD-MBD-GL-Update.pdf. Accessed 2 Apr 2024. 10.1016/j.kisu.2017.04.001 PMC6340919 30675420

[27] Ministry of Health, Labour and Welfare, Revision of Precautions. Zoledronic acid hydrate (preparations indicated for osteoporosis). [https://www.pmda.go.jp/files/000249928.pdf]. Accessed 2 Apr 2024.

[28] Ministry of Health Labour, and Welfare, Revision of Precautions. Sodium risedronate hydrate. [https://www.pmda.go.jp/files/000249931.pdf]. Accessed 2 Apr 2024.

[29] Ministry of Health, Labour and Welfare. Revision of Precautions. Minodronic acid hydrate. [https://www.pmda.go.jp/files/000249930.pdf]. Accessed 2 Apr 2024.

[30] Ministry of Health, Labour and Welfare. Revision of Precautions. Ibandronate sodium hydrate. [https://www.pmda.go.jp/files/000249926.pdf]. Accessed 2 Apr 2024.

[31] Ministry of Health, Labour and Welfare. Revision of Precautions. Etidronate disodium. [https://www.pmda.go.jp/files/000249927.pdf]. Accessed 2 Apr 2024.

[32] Ministry of Health, Labour and Welfare. Revision of Precautions. Alendronate sodium hydrate. [https://www.pmda.go.jp/files/000249925.pdf]. Accessed 2 Apr 2024.

[33] Act on the Pharmaceuticals and Medical Devices Agency (Act No.192 of 2002) [in Japanese]. [https://elaws.e-gov.go.jp/document?lawid=414AC0000000192]. Accessed 2 Apr 2024.

[34] Pharmaceuticals and Medical Devices Agency (PMDA), Pharmacoepidemiological studies for drug safety assessment under MIHARI framework (Japanese only). [https://www.pmda.go.jp/safety/surveillance-analysis/0045.html]. Accessed 2 Apr 2024.

