# Supplementary figures and images for: Increased risk of hypocalcemia with decreased kidney function in patients prescribed bisphosphonates based on real-world data from the MID-NET® in Japan: a new-user cohort study
Source: BMC Nephrol. 2024 Apr 15;25:134. doi: 10.1186/s12882-024-03553-7 (PMC11017550; doi:10.1186/s12882-024-03553-7)

## Additional file 1: Figure S1 Design diagram

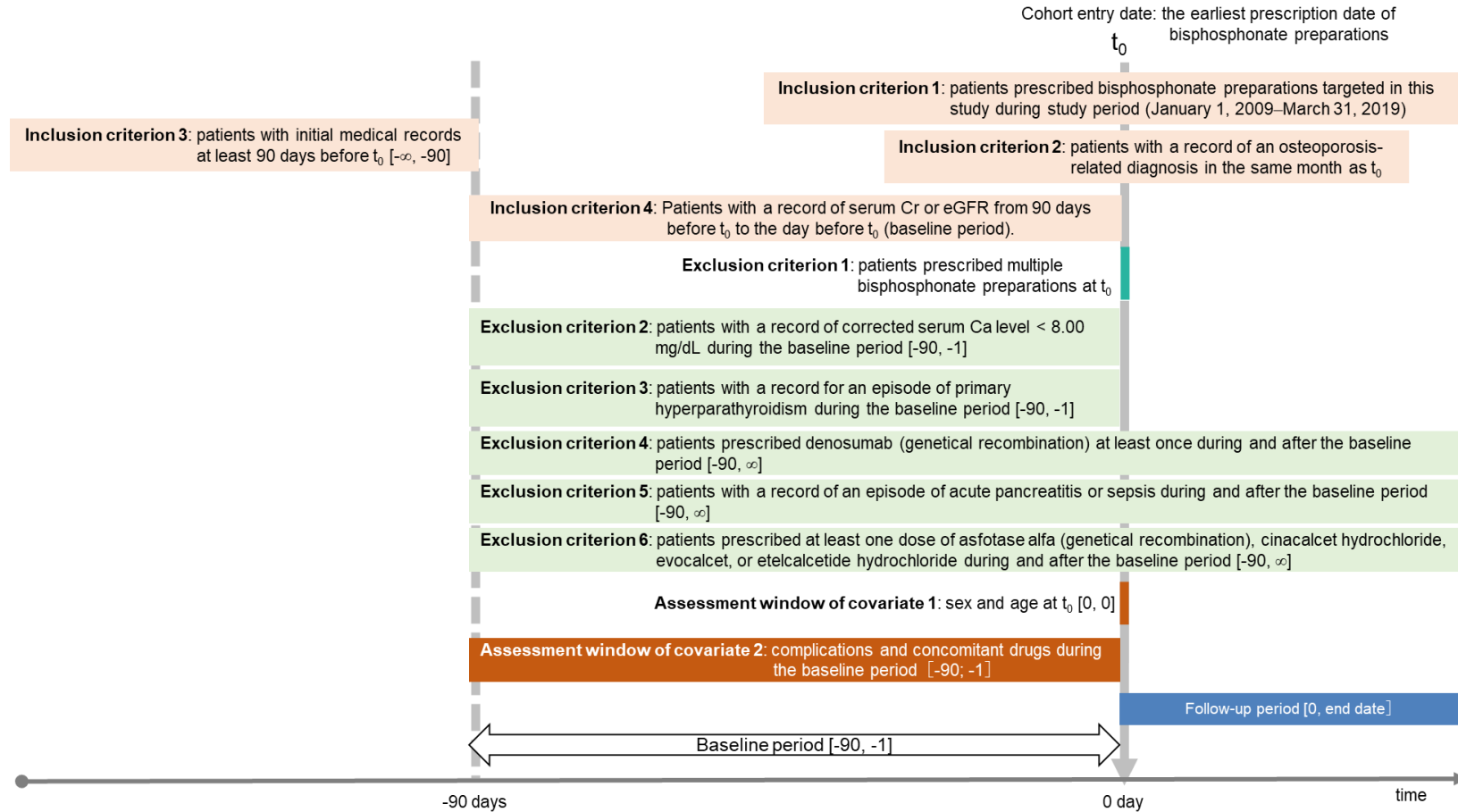

Supplement: Supplementary file 1 — Supplementary Material 1. [file 12882_2024_3553_MOESM1_ESM.pdf]
