# Supplementary material for: Increased risk of hypocalcemia with decreased kidney function in patients prescribed bisphosphonates based on real-world data from the MID-NET® in Japan: a new-user cohort study
Source: BMC Nephrol. 2024 Apr 15;25:134. doi: 10.1186/s12882-024-03553-7 (PMC11017550; doi:10.1186/s12882-024-03553-7)
Supplement: Supplementary file 2 — Supplementary Material 2. [file 12882_2024_3553_MOESM2_ESM.pdf]

Additional file 2: Table S1 Hazard ratios for hypocalcemia in the subgroup analysis on each bisphosphonate preparation (vs normal group)

Active Ingredient	Category	Number of patients[a]	Follow-up period (patient-year)	Number of cases[a]	Incidence rate[a] [95%CI] (/patient-year)		cHR[b] [95%CI]		aHR[b] [95%CI]	
Alendronate sodium hydrate	Normal	1,390	283.57	<10	<0.035	-	reference		reference	
	Mild	4,120	1,141.09	17	0.015	[0.008-0.022]	1.32	[0.44-3.94]	1.66	[0.55-5.00]
	Moderate	2,192	598.27	11	0.018	[0.007-0.029]	1.63	[0.52-5.12]	2.31	[0.70-7.69]
	Severe	270	50.20	10	0.199	[0.076-0.322]	13.53	[4.24-43.16]	16.03	[4.68-54.96]
Etidronate disodium	Normal	<18	1.61	0	-	-	reference		reference	
	Mild	11	0.83	0	-	-	-	-	-	-
	Moderate	<10	0.28	0	-	-	-	-	-	-
	Severe	0	0.00	0	-	-	-	-	-	-
Ibandronate sodium hydrate	Normal	25	10.67	0	-	-	reference		reference	
	Mild	56	25.83	0	-	-	-	-	-	-
	Moderate	34	24.52	0	-	-	-	-	-	-
	Severe	<10	0.56	0	-	-	-	-	-	-
Minodronic acid hydrate	Normal	387	253.66	<10	<0.039	-	reference		reference	
	Mild	1,277	925.26	<10	<0.011	-	1.14	[0.13-10.23]	1.09	[0.12-10.31]
	Moderate	608	388.21	<10	<0.026	-	1.27	[0.12-13.98]	1.15	[0.09-14.61]
	Severe	60	33.80	<10	<0.296	-	29.23	[3.26-261.83]	40.21	[3.75-430.64]
Sodium risedronate hydrate	Normal	781	201.63	<10	<0.050	-	reference		reference	
	Mild	2,098	700.87	<10	<0.014	-	1.50	[0.17-13.45]	1.78	[0.19-16.36]
	Moderate	1,050	340.66	<10	<0.029	-	2.22	[0.23-21.36]	2.57	[0.24-27.08]
	Severe	82	11.52	<10	<0.868	-	24.38	[2.20-269.82]	23.03	[1.89-280.35]
Zoledronic acid hydrate	Normal	<10	3.87	0	-	-	reference		reference	
	Mild	51	32.35	<10	<0.309	-	-	-	-	-
	Moderate	<35	22.49	0	-	-	-	-	-	-
	Severe	<10	3.49	0	-	-	-	-	-	-

aHR: adjusted hazard ratio, cHR: crude hazard ratio, CI: confidence interval

[a] When the number of patients was <10, an aggregated value was presented based on the MID-NET® publication rule, so that a specific number could not be identified.

[b] aHR and cHR were calculated based on the Cox proportional hazards model and aHR was adjusted with the covariates (see "Methods")
